# *Chiranthodendron pentadactylon* Larreat (*Sterculiaceae*), a Potential Nephroprotector against Oxidative Damage Provoked by STZ-Induced Hyperglycemia in Rats

**DOI:** 10.3390/plants12203572

**Published:** 2023-10-14

**Authors:** Eira Santiago-Balmaseda, David Segura-Cobos, María Eugenia Garín-Aguilar, Rubén San Miguel-Chávez, José Melesio Cristóbal-Luna, Eduardo Madrigal-Santillán, Gabriel Alfonso Gutierrez-Rebolledo, Germán Alberto Chamorro-Cevallos, Ricardo Pérez-Pastén-Borja

**Affiliations:** 1 Laboratorio de Toxicología Molecular, Escuela Nacional de Ciencias Biologicas, Campus Unidad Profesional Adolfo Lopez Mateos, Instituto Politecnico Nacional, Ave. Wilfrido Massieu w/n and Closed Street Manuel Stampa, Col. Industrial Vallejo, Mexico City 07700, Mexico; eirasantiagocbts@gmail.com; 2 Laboratorio de Amibas Anfizoicas, Facultad de Estudios Superiores Iztacala, Universidad Nacional Autonoma de México. Ave. Barrios 1, Col. Reyes Iztacala, Tlalnepantla de Baz 54090, Mexico; seguracd@unam.mx; 3 Laboratorio de Farmacobiología, Facultad de Estudios Superiores Iztacala, Universidad Nacional Autonoma de México. Ave. Barrios 1, Col. Reyes Iztacala, Tlalnepantla de Baz 54090, Mexico; maragarin@yahoo.com; 4 Posgrado en Botanica, Campus Montecillo, Colegio de Postgraduados, Mexico-Texcoco Highway Km 35.6, Texcoco Estado de México 56230, Mexico; sanmi@colpos.mx; 5 Laboratorio de Toxicologia de la Reproduccion, Escuela Nacional de Ciencias Biologicas, Campus Unidad Profesional Adolfo Lopez Mateos, Instituto Politecnico Nacional, Ave. Wilfrido Massieu w/n and Closed Street Manuel Stampa, Col. Industrial Vallejo, Mexico City 07700, Mexico; jcristoball@ipn.mx; 6 Laboratorio de Medicina de Conservación, Escuela Superior de Medicina, Campus Unidad Profesional Casco de Santo Tomas, Instituto Politécnico Nacional, Ave. Salvador Díaz Mirón w/n and Closed Street Plan de San Luis, Col. Miguel Hidalgo, Mexico City 11340, Mexico; eomsmx@yahoo.com.mx

**Keywords:** *Chiranthodendron pentadactylon*, antioxidant, nephrotoxicity, experimental diabetes, stress, traditional medicine Meso- and Southern America

## Abstract

Background: *Chiranthodendron pentadactylon*, known in Mexico as the “tree of the little hands”, flower’s infusion is used to treat kidney failure associated with diseases such as diabetes. The aim of this work is to evaluate the antioxidant effect of the methanolic extract of its flowers on oxidative damage in kidneys caused by streptozotocin in rats. Methods: The extract phytochemical profile was performed with HPLC. Antioxidant potential in vitro was determined with DPPH and total phenolic tests; antioxidant evaluation in vivo was performed in diabetic rats administered daily via the intragastric route (100 and 200 mg/kg) for 6 weeks; serum glucose/creatinine, food/water consumption, and urinary volume were measured. Relative weight, protein/DNA ratios and oxidative stress were measured in renal tissue. Results: The extract showed 20.53% of total phenolic content and IC_50_ of 18.05 µg/mL in DPPH, and this was associated with ferulic acid, phloretin and α-amyrin. Both doses showed a moderate decrease in the protein/DNA ratio in renal tissue, and the same behavior was observed for total urinary protein loss and serum creatinine, while the best antioxidant effect was exerted by a lower dose, which increased catalase activity and decreased lipid peroxidation in the kidneys. Conclusions: Results demonstrated that *C. pentadactylon* methanolic flower’s extract improves renal function through antioxidant mechanisms during experimental diabetes.

## 1. Introduction

Diabetic nephropathy is one of the most common consequences of diabetes; in the last 15 years, its prevalence has risen from 19% to 30%, with a global prevalence of 1016 per million diabetics [1]. In this context, the prevalence and incidence of DN continue to rise globally, since it is one of the leading causes of death associated with this disease [2,3].

Hyperglycemia during the early stages of diabetes promotes oxidative stress (OS) and a chronic inflammatory state that is initially of low impact, but its contribution increases progressively. Both mechanisms are key to the systemic damage produced by this disease. In the kidneys, it damages the microvasculature, impairing glomerular filtration and causing proteinuria in most patients, with the latter being the first clinical manifestation of DN. A total of 40% of patients with DN progress to end-stage renal disease, even though glycemic control, among other therapeutic actions, improves the patient’s life expectancy; end-stage renal failure continues to be one of the main complications associated with mortality due to diabetes. Current allopathic therapy available to treat diabetes only focuses on reducing hyperglycemia and last-generation drugs intended to restore kidney function during diabetes have poor accessibility, high cost and can generate adverse side effects such as fluid retention and heart failure [4].

In this context, the Mexican population still continues to use medicinal plants as an alternative treatment for these illnesses due to their easy access and low cost; various studies have shown the antioxidant and anti-inflammatory effectiveness of compounds obtained from medicinal plants, which have been studied as a cost-effective treatment for managing patients with chronic illnesses in most developing countries, as they are easily accessible and affordable [5,6,7]. In traditional Mexican medicine, *Chiranthodendron pentadactylon* Larreat—commonly known as the “Devil’s or monkey’s hand tree”, or “Mexican hand tree” in English, the “árbol de las manitas” (tree of the little hands) in Spanish, and in the native languages of Mexico such as nahuatl as “mācpalxōchitl” (palm flower) and “Canak or Canac” in the Mayan language—is distributed in the nation’s southwest states such as Guerrero, Oaxaca and Chiapas, but it is also cultivated in the central states of Morelos and Michoacán [8].

It has been extensively used since pre-Hispanic times in Meso- and Central America and in the treatment of secondary adverse physiological consequences provoked by chronic diseases in vital organs, for example, after heart strokes and during metabolic disorders such as diabetes, mostly as a remedy to renal end-stage complications by reducing edema (water retention) and regulating high blood pressure as a diuretic, as well to decrease serum cholesterol levels, with all of them related to low-impact oxidative and inflammatory processes [9,10,11,12]. *Chiranthodendron pentadactylon* Larreat flowers can still be found sold in markets in Mexico and Guatemala as a common herbal remedy for diabetes affectations such as kidney failure, where it is prepared in infusions using a tablespoon of -four sliced flowers (approximately 10 g) in 500 mL of hot water without boiling, for 10 min, then filtered and taken twice a day for a week [13].

It has been described that its flower’s high polarity extracts have showed antioxidant [13], antiviral [14], antiprotozoal [15], antidiarrheal [16] and anti-inflammatory [17] activities, which authors associate with its high content of phenolic compounds, secondary metabolites (SM) such as free flavonoids ((-)-epicatechin, catechin, isoquercitrin, astragalin) and finally glycosidic flavonoids such as tiliroside, which were isolated and tested as well [18].

However, to date, the possible beneficial effects of *C. pentadactylon* flower’s extract as an antioxidant agent in DN during experimental diabetes in laboratory animals has not been evaluated; therefore, in this study, the antioxidant effect of the methanolic extract of flowers from *C. pentadactylon* was evaluated on oxidative damage in kidneys caused by streptozotocin (STZ)-induced diabetes in rats.

## 2. Results

### 2.1. Phytochemical of Methanolic Extract of Chiranthodendron pentadactylon Flowers (MECP) Profile

**Figure 1:**
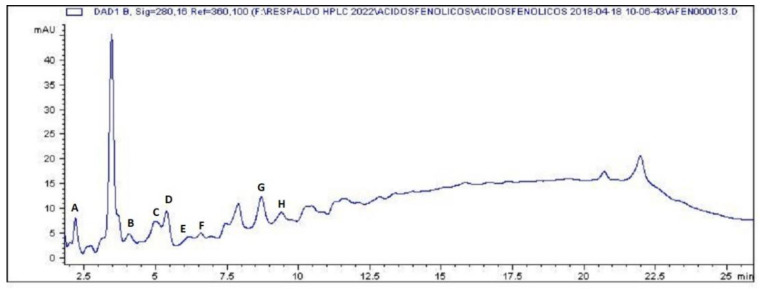
HPLC profile of phenolic acids identified and contained in MECP. (A) gallic acid, (B) chlorogenic acid, (C) syringic acid, (D) vanillic acid, (E) p-hydroxybenzoic acid, (F) caffeic acid, (G) ferulic acid and (H) p-coumaric acid.

Final yield of the extract was 31.4% (42.39 g) with respect of the initial total dried flower weight, from which chromatograms showed the presence of all pure standards tested for phenolic acids in MECP, with gallic acid (0.99 mg/g MECP, retention time [Rt] 2.10 min) being the first one, followed by chlorogenic acid (3.67 mg/g MECP, Rt 4.29 min), syringic acid (1.84 mg/g MECP, Rt 5.10 min), vanillic acid (2.46 mg/g MECP, Rt 5.38 min), p-hydroxybenzoic acid (traces, Rt 5.71 min), caffeic acid (2.10 mg/g MECP, Rt 6.64 min), ferulic acid (6.78 mg/g MECP, Rt 9.22 min) and p-coumaric acid (1.23 mg/g MECP, Rt 9.76 min) (Figure 1).

**Figure 2:**
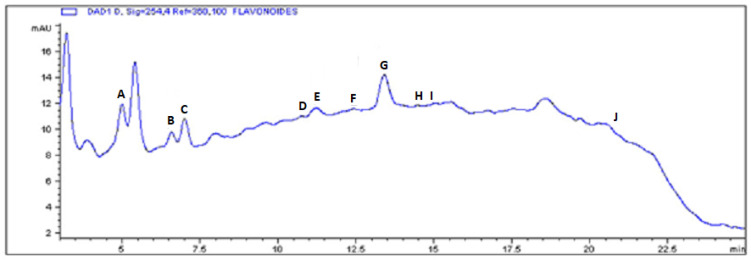
HPLC profile of identified flavonoids present in MECP. (A) rutin, (B) phlorizin, (C) myricetin, (D) luteolin, (E) quercetin, (F) naringenin, (G) phloretin, (H) apigenin, (I) kaempferol and (J) galangin.

Chromatograms for flavonoid quantification exhibited four concordances for all the tested pure standards in MECP with these SMs, with a major presence of rutin (traces, Rt 4.40 min), phlorizin (0.59 mg/g MECP, Rt 6.78 min), myricetin (traces, Rt 7.49 min), luteolin (traces, Rt 11.18 min), quercetin (0.14 mg/g MECP, Rt 10.97 min), naringenin (1.97 mg/g MECP, Rt 12.27 min), phloretin (3.40 mg/g MECP, Rt 13.10 min), as well as traces of other flavonoids such as apigenin (Rt 14.66 min), kaempferol (Rt 14.77 min) and galangin (Rt 22.17 min) (Figure 2).

**Figure 3:**
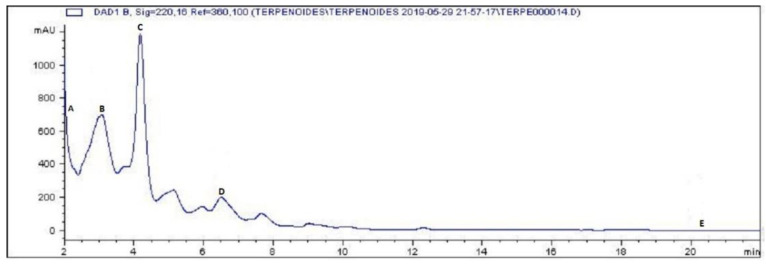
HPLC profile of identified terpenoids in MECP. (A) carnosol, (B) stigmasterol, (C) oleanolic acid, (D) α-amyrin and (E) β-sitosterol.

Finally, chromatograms made for the identification of terpenoids in MECP showed a match for three of these SMs, traces of carnosol (Rt 2.34 min), followed by stigmasterol (22.33 mg/g MECP, Rt 4.29 min), traces of oleanolic acid (Rt 4.27 min), α-amyrin (101.54 mg/g MECP, Rt 6.44 min) and β-sitosterol (0.17 mg/g MECP, Rt 20.37 min) (Figure 3).

Of all identified SMs, who were compared with pure standards through HPLC, the total quantity summations of each type showed that MECP obtained from its flowers has a higher amount of terpenoids (124.04 mg/g MECP), followed by phenolic acids (19.16 mg/g MECP) and flavonoids (6.10 mg/g MECP).

### 2.2. Antioxidant Potential In Vitro

TCP results showed values of 205.27 mg eq GA/g of the extract at 0.1 mg/mL of the tested concentration, which represents up to 20.53% of the total dry weight of the extract, while it exerted a significant scavenging capacity on the DPPH radical with an IC_50_ value of 18.05 µg/mL for MECP, which is lower compared to pure quercetin, and had an IC_50_ value of 5.92 µg/mL. These results are consistent with those shown in the chemical composition analysis of MECP, since SMs such as phenolic acids and flavonoids were identified.

### 2.3. MECP Effect on Glycemia, Total Body Weight and Urinary Volume

All altered parameters of the clinical evaluation of animals, as well as the analytes of blood biochemistry, showed no improvement caused by treatments during the development of experimental diabetes, and are summarized in Table 1. Before STZ induction, all animals presented a normal glycemic state ≈90 mg/dL. Seventy-two hours after STZ injection, diabetic-induced groups showed a significant increase (four-fold) in plasma glucose (≈400 mg/dL) compared to the values showed by healthy rats of vehicle and E200 group (≈100 mg/dL). At the end of the experiment, similar values were maintained 6 weeks prior, without observable differences between the treated groups and the DG-untreated group at final day (≈500 mg/dL) compared to both groups of healthy rats (79 ± 4.63 mg/dL). A consistent total BW was seen for non-hyperglycemic animals of vehicle and E200 groups (≈360 g) at the experiment endpoint day. Nonetheless, total BW showed a significant decrease of almost ≈25% in all other four diabetic-induced groups, even in those that received treatments (Table 1).

Food and water ingestion increased statistically in a four-fold manner in all the diabetic rat groups (≈40 g/24 h, and ≈150 mL/24 h, respectively), including those treated with vitamin E and MECP and compared to the results showed by both healthy animal groups (≈10 g/24 h, and ≈25 mL/24 h, respectively). Also, urinary volume was robustly significantly increased in a ten-fold manner in groups with experimental diabetes (≈90 mL/24 h), even in those that were administered with the antioxidant reference and MECP, compared to the data shown by control healthy rats groups (≈10 mL/24 h) (Table 1).

### 2.4. Renal Relative Weight and Protein/DNA Ratios during Experimental Diabetes

Kidney weight/body weight index results showed a significant increase in diabetic-untreated animals (4.91 ± 0.25) compared to healthy vehicle-treated rats (2.81 ± 0.03) and to healthy rats administered with MECP at 200 mg/kg (2.86 ± 0.1).

**Figure 4:**
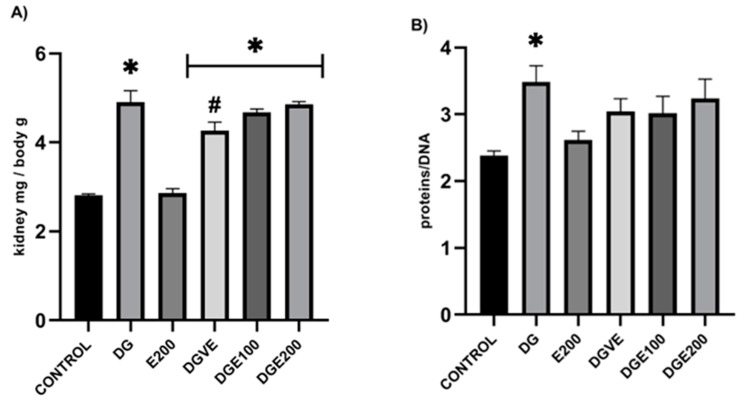
Effect of MECP in renal relative weight of STZ-induced diabetic rats. (**A**) renal weight mg/BW g ratio at 6 weeks; (**B**) protein/DNA ratio. Data are shown as mean ± SEM (n = 6). Analyzed with ANOVA followed by a Tukey post hoc test with ∗ statistical significance (*p* < 0.05) vs. vehicle group, # statistical significance (*p* < 0.05) vs. *diabetic group* (DG); DNA, desoxyribonucleic acid; MECP, methanolic extract of *C. pentadactylon*; E200, healthy animals + MECP 200 mg/kg BW; DG, diabetic-untreated animals; DGVE, diabetic animals + vitamin E 250 mg/kg BW; DGE100, diabetic animals + MECP 100 mg/kg BW; DGE200, diabetic animals + MECP 200 mg/kg BW.

In contrast, diabetic animals treated with vitamin E exerted a significant reduction in this index (4.27 ± 0.19); for diabetic rats that received MECP treatments at 100 and 200 mg/kg, no differences were observed (4.67 ± 0.08, and 4.85 ± 0.06, respectively), with them being statistically similar to the DG group (Figure 4A).

On the other hand, a significant increase in protein/DNA ratio was also shown in the DG group (3.49 ± 0.24) versus the vehicle (2.38 ± 0.06) and E200 (2.62 ± 0.13) of the healthy rat groups. Treatments (vitamin E and MECP) given to diabetic animals did not change these values, as there were no significant differences between them and the other three groups (3.10 ± 24) (Figure 4B).

### 2.5. Kidney Function in Diabetic Rats

**Figure 5:**
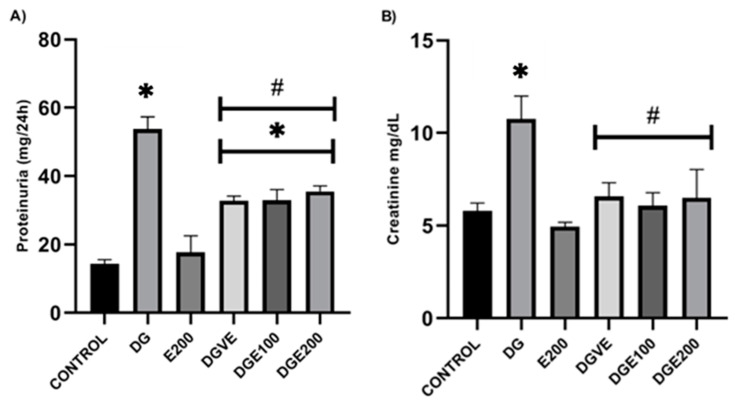
MECP effect in kidney function of STZ-induced diabetic rats. (**A**) proteinuria in 24 h; (**B**) serum creatinine. Data are shown as mean ± SEM (n = 6). Analyzed with ANOVA followed by a Tukey post hoc test with ∗ statistical significance (*p* < 0.05) vs. vehicle group; # statistical significance (*p* < 0.05) vs. *diabetic group* (DG). MECP, methanolic extract of *C. pentadactylon*; E200, healthy animals + MECP 200 mg/kg BW; DG, diabetic-untreated animals; DGVE, diabetic animals + vitamin E 250 mg/kg BW; DGE100, diabetic animals + MECP 100 mg/kg BW; DGE200, diabetic animals + MECP 200 mg/kg BW.

Proteinuria results showed a significant increase in the total urinary protein loss/24 h (53.84 ± 3.54 mg/24 h) in the DG group compared to the results observed in the vehicle group (14.38 ± 1.19 mg/24 h), and there was no differences between the E200 healthy animal groups (17.71 ± 4.89 mg/24 h). Moreover, in diabetic rats treated with VE (250 mg/kg) and MECP at 100 and 200 mg/kg, a significant reduction in proteinuria/24 h in almost ≈38% occurred compared to diabetic-untreated group (53.84 ± 3.54 mg/24 h) (Figure 5A).

Regarding serum creatinine, the DG group showed a higher concentration (10.76 ± 1.23 mg/dL) compared to vehicle and the E200 healthy animal groups (5.8 ± 0.42, and 4.95 ± 0.23 mg/dL, respectively), and there were no statistical differences between these two. On the other hand, diabetic-treated rat groups showed a significant reduction of serum creatinine levels of almost ≈40% compared to results of diabetic-untreated rats (10.76 ± 1.23 mg/dL); however, values such as these (5.8 ± 0.42 mg/dL) were exerted by healthy rats that only received vehicles (Figure 5B).

### 2.6. Antioxidant Enzyme Activity in Diabetic Rats’ Renal Tissue

**Figure 6:**
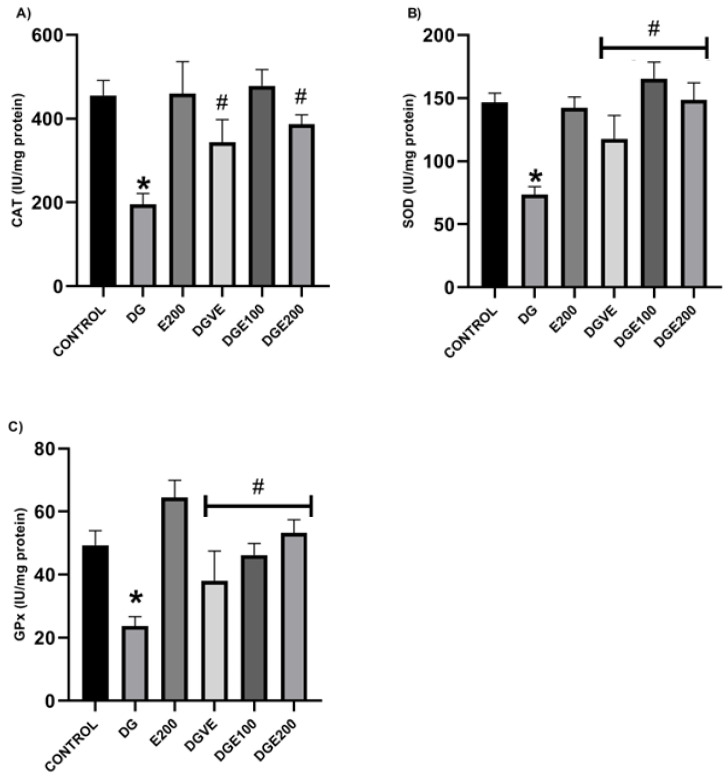
Effect of MECP in antioxidant enzymes activity during experimental diabetes induced with STZ in rats: (**A**) catalase, (**B**) SOD, (**C**) GSH-Px. Data are shown as mean ± SEM (n = 6). Analyzed with ANOVA followed by a Tukey post hoc test with * statistical significance (*p* < 0.05) vs. vehicle group; # statistical significance (*p* < 0.05) vs. diabetic group. CAT, catalase; SOD, superoxide dismutase; GPx, glutathione peroxidase; MECP, methanolic extract of *C. pentadactylon*; E200, healthy animals + MECP 200 mg/kg BW; DG, diabetic-untreated animals; DGVE, diabetic animals + vitamin E 250 mg/kg BW; DGE100, diabetic animals + MECP 100 mg/kg BW; DGE200, diabetic animals + MECP 200 mg/kg BW.

Activity of three enzymes (CAT, SOD and GSH-Px) in diabetic-untreated rats decreased significantly compared to the healthy rats group administered only with vehicles, and there were no statistical differences between this group and healthy rats that received MECP at 200 mg/kg (Figure 6).

In the case of CAT enzyme, the DGE100 experimental diabetic animals presented an almost three-fold increased activity after the administration treatment period, compared to the results shown by diabetic-untreated rats (195.58 ± 25.5 IU/mg), restoring activity to the values observed in healthy rats from the vehicle (455.45 ± 35.87 IU/mg) and E200 (459.6 ± 76.6 IU/mg) groups. On the other hand, diabetic rats treated with vitamin E at 250 mg/kg and MECP at 200 mg/kg showed both a statistical different increase in CAT activity and in the renal tissues of almost ≈two-fold when compared to the DG-untreated group; however, both treatments were unable to restore it to normal values (Figure 6A).

In addition to this scenario, SOD’s activity was also significantly lower in diabetic rats that were administered only with the vehicles (73.45 ± 6.33 IU/mg) vs. not-induced rats that treated with the vehicles (146.6 ± 7.3 IU/mg). There were no differences between this late group and the E200 (142.23 ± 8.7 IU/mg). In this context, the treatment with MECP in diabetic groups, DGE100 and DGE200, restored this antioxidant enzyme activity ≈two-fold, even reaching values exerted by the healthy rats in both control groups, although SOD’s function in kidneys of diabetic rats was upregulated with vitamin E administration in 60% of cases compared to diabetic rats without treatment, and its renal tissue concentrations were not normalized to basal levels shown by healthy rats (Figure 6B).

Finally, as it happened to other two antioxidant enzymes, the GSH-Px activity significantly decreased in the diabetes-untreated animals group without treatment (23.75 ± 2.96 IU/mg) compared to the results observed in healthy rats of both vehicle (49.32 ± 4.63 IU/mg) and E200 (64.52 ± 5.46 IU/mg) control groups, with no statistical differences between them; while treatments generated in diabetic animals’ renal tissues showed an increase in GHS-Px activity of 58% for those that were administered with vitamin E, and in a dose-dependent manner for the diabetic rats treated with MECP at 100 and 200 mg/kg with increments in its activity of 90% (*p* < 0.05) and 120% (*p* < 0.01), respectively (Figure 6C).

### 2.7. LPO Rate in Renal Tissue

**Figure 7:**
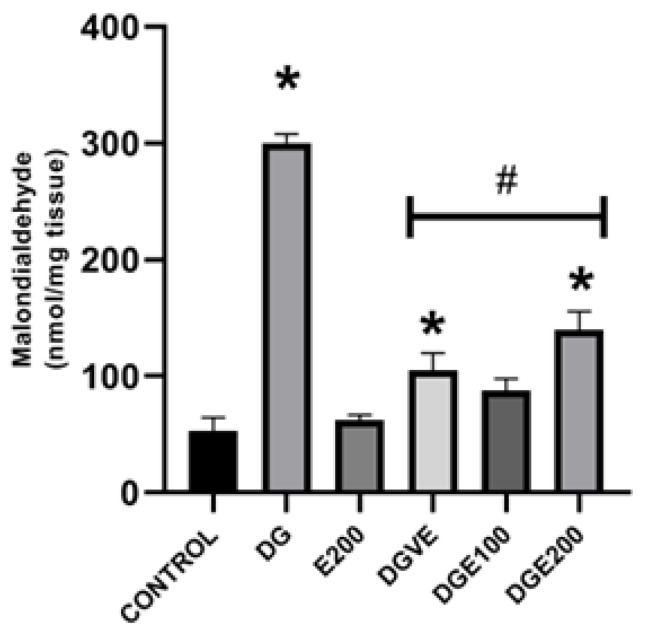
Effect of MECP in lipid peroxidation rate in renal tissue of diabetic rats. Data are shown as mean ± SEM (n = 6). Analyzed with ANOVA followed by a Tukey post hoc test with * statistical significance (*p* < 0.05) vs. vehicle group; **#** statistical significance (*p* < 0.05) vs. diabetic group. MDA, malondialdehyde; MECP, methanolic extract of C. pentadactylon; E200, healthy animals + MECP 200 mg/kg BW; DG, diabetic-untreated animals; DGVE, diabetic animals + vitamin E 250 mg/kg BW; DGE100, diabetic animals + MECP 100 mg/kg BW; DGE200, diabetic animals + MECP 200 mg/kg BW.

All these previous modifications in renal tissues’ antioxidant environment of diabetic rats were supported, and the results observed for LPO rate of these renal samples were corroborated. MDA determination showed that there was a significant increase in LPO in diabetic-untreated rats’ kidneys (300.17 ± 7.8 nmol/mg) compared to the results shown by healthy rats administered only with the vehicles (53.16 ± 11.38 nmol/mg). Moreover, it was observed that there was no statistical difference between the vehicle and E200 control groups (62.02 ± 4.69 nmol/mg) (Figure 7); meanwhile, treated groups showed a significant decrease in the LPO rate in renal tissue of 65% administered with vitamin E, followed by a reduction of 53% for diabetic rats administered with MECP at 200 mg/kg and compared to the rats of diabetes-untreated control group; however, the diabetic animals that received MECP at 100 mg/kg showed a statistical decrease in the LPO rate in kidneys of 71% compared to diabetes-untreated group, and reached values comparable to those of the healthy rats in the vehicle group (Figure 7).

## 3. Discussion

In this work, the antioxidant effect of *Chiranthodendron pentadactylon* flowers on altered kidney function during an STZ-induced diabetes 1 model in male Wistar rats was assessed. This specimen was chosen to being evaluated because of its wide use in traditional Mexican medicine for the treatment of late adverse sequelae caused, on organs such as the kidney, by the oxidative damage characteristic of the pathophysiology of chronic degenerative diseases such as diabetes [17,18,19,20]; however, this is the first evidence of the beneficial contribution through its antioxidant effect of this specie in DN, on one of the greatest complications suffered by patients with diabetes, through the regulation of renal function by decreasing the OS generated with hyperglycemia, supporting its ethnomedicinal use of also regulating blood pressure, and possibly treating diabetes from other action mechanisms different than hypoglycemia [11,13,21].

After chemical fragmentation of MECP, preliminary chemical composition screening was positive for phenolic compounds, most importantly, and studied SMs with antioxidant properties [22], which are well-known for their direct inhibition of the chemical structures of free radicals. The beneficial mechanism proposed for MECP may be similar to those described in previous studies with other medicinal plants with high TPC, such as *Nelumbo nucifera* (lotus flower) [5] and *Camelia sinensis* (green tea) [23], where its nephroprotective effect was correlated to its antioxidant effect against oxidative damage provoked by experimental diabetes in vivo.

For in vitro antioxidant potential assays, it is understood that the lower the IC_50_ value, the greater the reduction capacity. However, it must be considered that positive controls (quercetin and gallic acid), against which MECP was compared in both in vitro techniques, are pure phenolic compounds, of whose antioxidant capacity has been widely verified [24]. Moreover, MECP is a complex mixture of not only polar compounds but apolar nature SMs as well. Furthermore, these results are consistent with those reported by Ibarra-Alvarado et al. [13], who demonstrated a positive correlation between TPC of the direct aqueous extract of the flowers of *C. pentadactylon* (221.4  ±  0.6 mg eq GA/g of extract) and its antioxidant potential in vitro, such as the direct inhibition of DPPH free radicals (IC_50_ =140.6 ± 1.0 µg/mL). Although there were lower values of TPC for MECP in this work, which had a preprocessing of degreasing with hexane, the DPPH IC_50_ value was greater for MECP in spite of that, even with the previous de-fatty process, compared to the direct aqueous extract reported by authors. In the same context, Villa-Ruano et al. [20] also reported an IC_50_ value for DPPH of 102.60 ± 6.60 µg/mL for the direct ethanolic extract of this specie’s shoots and leaves, which is a value greater than that observed for MECP.

Although there are few studies that evaluate the antioxidant potential in vitro of extracts from this species, there are studies where the majority of its SMs have been determined in DPPH inhibition as syringic acid (IC_50_ = 1.5 µg/mL, [25]), vanillic acid (20 µm inhibits almost 30%, [26]), ferulic acid (IC_50_ = 1 µm, [27]), phlorizin (IC_50_ = 10 mM, [28]), phloretin (IC_50_ = 0.6 mmol/L, [29]) and α-amyrin (IC_50_ = 10 mM, [30]). Hence, the notorious value of IC_50_ shown by the MECP on the radical DPPH is due to the majority presence of these SMs identified in MECP using the HPLC technique.

In relation to in vivo results, STZ, due to its similarity to glucose molecule, enters β cells of the pancreas through the GLUT-2 transporter and produces cellular necrosis in the pancreas. Consequently, additionally to this damage, insulin production by these cells is altered, and lost within the time that causes hyperglycemia [31]. One of the most important inclusion parameters when studying experimental diabetes in rodents is that they have a blood glucose level of >300 mg/dL at 72 h after STZ administration, provoking 6-week-maintained hyperglycemia throughout the experiment, along all classic manifestations of this illness comprising polydipsia, polyphagia, polyuria and loss of body weight [32], which were not modified daily with MECP for the 6-week administration period in this study.

By analyzing the protein/DNA ratio, a mild improvement could be attributed to MECP and vitamin E treatments during experimental diabetes by reducing this preliminary parameter of a possible renal hypertropia. Although the kidney’s relative weight and its protein mg/DNA ratio, together with a dysregulation of renal function demonstrated by serum creatinine elevation and proteinuria in STZ-induced diabetic rats, are important to consider as indicators of a possible and incipient renal hypertrophy, as described by previous studies [33], it is necessary to perform specific histological analyses on renal tissue in subsequent experiments to ensure that treatment with MECP is able to reduce renal hypertrophy and avoid the appearance of kidney edema associated with a decrease in the organ-relative weight ratio [6].

Among other aspects, DN develops concomitantly along with hyperglycemia, and the severity and development of the disorder to a chronic stage is reflected in the biochemical parameters of proteinuria and serum creatinine, with them both being useful in experimental models of diabetic animals induced with STZ, and in clinic studies helping to assess early diagnosis of kidney injury in patients with diabetes 1 [31], and are indicators of improvement when it is reversed by new nephroprotective molecules [21].

It is well known that chronic hyperglycemia depletes kidney function and favors the appearance of renal oxidative damage caused by a sustained perpetual OS microenvironment. Late-stage diabetes is also characterized by the oxidative inflammatory pathways’ activation, kidney’s altered function of antioxidant enzymes, and exacerbated renal tissue’s LPO [34,35,36].

As evidence of the relationship that exists between OS and kidney damage, in this work, proteinuria, high serum creatinine and an increase in the relative weight ratio were documented in diabetic-untreated rats as possible processes of established renal OS and inflammation. Nevertheless, it is essential to evaluate specific biomarkers of inflammation to elucidate this issue. Although it is known that the STZ model is used to experimentally emulate diabetes, in the search for new hypoglycemic agents, previous work has described the use of the model to evaluate the antioxidant activity of new molecules against OS associated with these chronic degenerative diseases, and its adverse sequelae, and how biological systems such as neuronal and renal are affected [36,37,38,39].

Due to this, authors who have studied the antioxidant effect of various substances, such as vitamins E and C [33], did not use a hypoglycemic reference drug, since it is not the mechanism by which it is desired to exert a possible protective effect on other tissues against OS caused by hyperglycemia. In this context, Niu et al. [40] demonstrated in a study of experimental STZ-induced diabetes in rats that the administration of *Eucommia ulmoides* root extract, although it did not decrease hyperglycemia, helped to improve renal integrity and function, without using a hypoglycemic agent as a control.

These nephroprotective effects of various medicinal plants have even been proved to occur independently of hypoglycemic activity [5], pointing out that phenolic compounds are responsible for the effect of inhibiting the formation of advanced glycation end products (AGEs) [41] and reactive oxygen species (ROS) during DN development of STZ-induced experimental diabetes in murine models [42].

These renal structural and function alterations were proportional to the antioxidant enzymes activity decrease and a higher quantity of MDA in the kidneys’ homogenates of diabetic-untreated rats; however, renal function parameters improve notoriously in diabetic rats treated with MECP (decreased proteinuria and serum creatinine), and this may be provoked by MECP’s high amounts of ferulic acid which can regulate creatinine renal depuration values by also decreasing OS damage in renal tissue [21].

Due to the lack of hypoglycemic activity induced with MECP, it can be inferred that any nephroprotective effect observed by treatments is unrelated to the catalytic pathway of glucose metabolism, and rather might work through a diuretic effect by one of its main SMs identified in this work, such as syringic acid [43], vanillic acid [44], ferulic acid [21] and α-amyrin [45], which can inhibit NF-kB. Cytokine is also related to high blood pressure (with this also being an ethnomedicinal use of the species), with a beneficial impact on the renal system during diabetes, such as the other identified SMs in previous works by other authors, such as (-)-epicatechin [16,46].

It is worth highlighting that the results compared to the 6-week treatment in this work were obtained by Kędziora-Kornatowska et al. [6], who demonstrated the progressive improvement of the kidneys’ antioxidant endogenous defense parameters by testing vitamin E and C effects on STZ-induced experimental DN, since week 6 to week 12 of the treatment; at day 42, authors identified an increase in antioxidant enzyme’s function. However, prevention of renal hypertrophy and complete improvement in kidney function were only significant after 12 weeks of both vitamin treatments. Similar results related to antioxidant enzymes activities were also reported previously for *C. pentadactylon* extract by Segura-Cobos et al. [33], in a STZ-induced DN in rats.

In this context, this work showed that MECP had a protective effect in the course of DN associated with STZ-induced diabetes, mostly through its antioxidant effect exerted by some of its phenolic compounds identified in *C. pentadactylon* flowers in previously published studies such as (-)-epicatechin [16], which is a catechin-isoflavonoid that could exert nephroprotection against OS provoked by diabetes in chronic stages through direct structure inhibition of free radicals. This was also demonstrated for other medicinal species such as the *Camellia sinensis* in chronic degenerative models in rats [47], as well as ferulic acid, and major SMs identified in MECP, which can improve antioxidant enzymes activity and prevent LPO of renal tissue [21]. However, the enhancing effect that certain SMs exert on the activity of antioxidant enzymes (CAT, SOD and GSH-Px), such as syringic acid [43], phloretin [48] and α-amyrin (inhibitor of cytochromes P_450_), reduces the number of ROS produced in tissues where these enzyme systems are abundant, such as the kidneys [49], and have been demonstrated in previous works, to which the effect generated by the MECP can be attributed. Therefore, it is likely that the renal regulation mechanism of MECP is triggered by its antioxidant effect, due to its high phenolic compounds composition, and thus prevents LPO and reestablishes the normal function of antioxidant enzymes at a renal level during experimental STZ-induced diabetes, even more than the treatment with vitamin E.

Efficiency and action mechanisms of vitamin E, as an antioxidant agent, have been widely documented, referring that it works by stimulating the activity of antioxidant enzymes and avoiding the translocation of inflammatory factors towards the nucleus, such as NF-kB [7,33,50,51]. MECP possibly has anti-inflammatory effect due to its SM’s NF-kB-inhibitors, such as syringic acid [43], and ferulic acid [21], which demonstrate this activity for in vitro tests, as well as vanillic acid [44] as well α-amyrin [45], which achieve NF-kB inhibition in acute and chronic inflammation with in vivo evaluation in mice models. Furthermore, although phloretin does not inhibit NF-kB, if it decreases TNF-α-stimulated gene expression of vascular adhesion proteins preventing leukocyte migration [52] it inhibits all of them, including the oxidative burst of inflammation as well the oxidative damage in renal tissue during experimental diabetes; however, other possible action mechanisms should be investigated further.

In the past, interest in new therapies for the treatment of diabetes was limited to hypoglycemic agents. Currently, it is known that, although the main source of damage in diabetic patients is sustained hyperglycemia, restoration of an organism’s antioxidant endogenous defenses is not only achievable with glycemia normalization, and that there are benefits of complementing glycemic management with antioxidant co-adjuvant therapies, which counteract oxidative damage mechanisms related to chronic complications of diabetes such as DN [21]. The foregoing provides consistent evidence of the benefit of antioxidant molecules as a complementary treatment to pharmacological control of the complications of this disease.

## 4. Materials and Methods

### 4.1. Plant Specimen

*Chiranthodendron pentadactylon* Larreat flowers were obtained from Mercado Pasaje Catedral located in República de Guatemala street #10, Col. Centro, PC, 06000, Mexico City (coordinates 19°26′3.403′′ N 99°7′31.173′′ W), in March of 2018. Those flowers that were complete, without color changes, completely formed and not in bud, that did not have spots or were contaminated by pests were cut off and selected for this study. Complete dried plant samples were deposited at the Herbario Iztacala Herbarium, identified as *Chiranthodendron pentadactylon* Larreat by Biologist Ma. Edith López Villafranco and were registered with the batch specimen number 2949 IZTA. Based on data described in the International Plant Names Index, entered through http://www.theplantlist.org/ (accessed on 20 July 2023), it was determined that this specie belongs to the taxonomic family of Sterculiaceae. (https://www.ipni.org/n/822543-1, accessed on 20 July 2023).

### 4.2. Obtention of Methanolic Extract of Chyrodendron pentadactylon flowers

Selected flowers were air-dried at room temperature (25 ± 5 °C) in dark conditions for three days, and then crushed with a mechanical mill until they became powder. Extraction of the powder of flowers (135 g) was performed with Soxhlet equipment (“IMPARLAB” brand, 50 mL, nozzles 55/50 and 24/40) [19] using 300 mL of hexane at 30 °C, and subsequently same volume of methanol at 60 °C for 24 h. Residue was filtered and concentrated at 40 °C using a rotary evaporator (Buchii RE-111, Buchii, Meierseggstrasse 40, 9230 Flawil, Switzerland) coupled to a vacuum system (BuchiiVacV-153, Buchii, Meierseggstrasse 40, 9230 Flawil, Switzerland) and a cooling system (ECO20, Atlas Copco Group, NASDAQ OMX Stockholm, ATCO A, ATCO B, Sweden.) and refrigerated at the end at 4 °C in dark conditions until its use. The extraction process was carried out to dryness under reduced pressure for the total elimination of alcohol. MECP was dissolved previously in propylene glycol (10%) in water, for in vivo experiment, because previous studies have shown that the possible nephrotoxic effect of this solvent agent is achieved in rats via oral administration of concentrated solutions of 45%, at doses of 1000 mg/kg, for periods of 28 to 90 days [53,54,55]. Although ethnomedicinal use of this specie is described as hydroalcoholic preparation or in infusion with water, it was chosen to make a methanolic extract to have greater chemical complexity in the extract and to be able to identify and quantify the greatest number of secondary metabolites present in flowers, mainly flavonoids, which are of high polarity, and have shown greater potential and antioxidant effect in previous studies.

### 4.3. Chemical Characterization of MECP with HPLC

Phytochemical analysis for the identification and quantification of the main secondary metabolites (SMs) contained in MECP was carried out with high performance liquid chromatography (HPLC) in Hewlett Packard liquid chromatograph (model 1100), equipped with an automatic injector Agilent Technologies (model 1200), and Hewlett Packard diode array detector (model 1100), and Hewlett Packard quaternary pump (model 1100). Management and programming of equipment, data registration and processing was performed using Agilent Technologies 2006 B.02.01 program (ChemStation Family Software Products version Rev B.02.01, 2006). All used standards showed 90 to 99% purity and were purchased at Sigma–Aldrich, St. Louis, MO, USA.

Experimental conditions used for phenolic acids search were Macherey-Nagel Nucleosil column (5 μm, 125 × 4.0 mm diameter i.d.), with a gradient of (eluent A) acidified water (H_2_0 pH 2.5) with TFA (trifluoroacetic acid) and (eluent B) CH_3_CN (acetonitrile). Other experimental parameters included the following: flow rate of 1 mL/min and injection volume of 20 µL; temperature at 30 °C; peaks were detected at wavelength of 280 nm; and analysis time of 23 min. Caffeic, gallic, chlorogenic, vanillic, ferulic, *p*-coumaric and syringic acids were used as pure standards patterns [56].

Experimental conditions used for flavonoids quantification were Hewlett Packard Hypersil ODS column (5 μm, 125 × 4.0 mm diameter i.d.), with a gradient of (eluent A) acidified water (H_2_0 pH 2.5) with TFA (trifluoroacetic acid) and (eluent B) CH_3_CN (acetonitrile). Other experimental parameters included the following: flow rate of 1 mL/min and injection volume of 20 µL; temperature at 30 °C; peaks were detected at wavelength of 254, 316 and 365 nm; and analysis time of 25 min. The standards used were rutin, phlorizin, myricetin, quercetin, naringenin, phloretin, apigenin and galangine [56].

Experimental conditions used for terpenoid identification were carried out with ZORBAX Eclipse XDB-C8 column (5 μm, 125 × 4.0 mm diameter i.d.). Isocratic analysis was performed using as eluent A CH_3_CN 80%, and as eluent B: H_2_O 20%, with the following experimental parameters: flow rate of 1 mL/min and injection volume of 20 µL; temperature at 40 °C; peaks were detected at wavelength of 215 and 220 nm; and analysis time of 21 min. Terpenoids carnosol, ursolic acid, stigmasterol, oleanolic acid, α-amyrin and β-sitosterol were used as pure standards [56].

### 4.4. In Vitro Antioxidant Potential Techniques

#### 4.4.1. Total Phenolic Content (TPC)

Folin–Ciocalteau method was executed using gallic acid as reference standard [57,58]. Each experiment was carried out in triplicate and results were described as mg equivalents of gallic acid (GA)/g of dried extract (mg eq GA/ g dried MECP extract). A calibration curve was performed using pure gallic acid as a standard diluted in deionized water, in a range of concentrations from 0.02 to 0.12 mg/mL (R^2^ = 0.99, y = 13.398x + 0.0013). Sample MECP was evaluated using a stock solution of 0.2 mg/mL in deionized water to carry out the assay, using 250 μL and adding deionized water until a final volume of 1 mL, after which 500 μL of Folin–Ciocalteau and 1.5 mL of sodium carbonate was added. Then, the reaction was left to incubate at room temperature for two hours. All absorbance was measured with a spectrophotometer (Shimadzu Double Beam Scanning UV–Vis (Model UV-1700)) and all samples were measured at 750 nm.

#### 4.4.2. DPPH Radical Inhibition

Antioxidant potential of MECP was also evaluated using in vitro DPPH (2,2-Diphenyl-1-picrylhydrazyl, Sigma–Aldrich, No. cat D9132) scavenging assay [59]. Free radical MECP was dissolved in methanol, and the last two were evaluated at the same concentrations (from 4 to 40 µg/mL, R^2^ = 0.99, y = 1.5967x + 21.187). Experiment was determined in triplicate, and inhibition potential was calculated with % inhibition = [AC-AS/AC] × 100, where AS: absorbance sample, AC: absorbance DPPH control. A calibration curve was performed using pure quercetin as a standard in a range of concentrations from 2 to 20 µg/mL (R^2^ = 0.99, y = 3.9877x + 34.381). All of absorbance was measured with a spectrophotometer (Shimadzu Double Beam Scanning UV–Vis (Model UV-1700, SHIMADZU Corporation, Nishinokyo Kuwabara-cho, Nakagyo-ku, Kyoto 604-8511, Japan)) at 515 nm. Finally, median inhibitory concentration (IC_50_) was calculated for MECP and quercetin. Results were reported as µg/mL after extrapolation on the calibration curve.

### 4.5. In Vivo Models

#### 4.5.1. Experimental Animal Conditions

Thirty-six adults male Wistar rats, body weight (BW) of 240 ± 10 g, were obtained from Facultad de Estudios Superiores Iztacala *vivarium*. For the purposes of this experimental model, the influence of biological sex on the results is negligible. Animals were located in plastic cages during a 7-day conditioning period in the Pharmacology Laboratory of the Interdisciplinary Research Unit in Health Sciences and Education; rats were maintained prior and during the experiments under the following laboratory-controlled room conditions: temperature (25 ± 2 °C), light/dark automatized cycles (12 h/12 h), humidity saturation (55–80%), and with daily changes of food (RodentChow^®^, PURINA Company, France) and filtered water. Measurements were performed in the consumption of both food and water during experimental protocol.

Procedures conducted on the laboratory animals followed the statutes of the International Committee for the Care and Use of Laboratory Animals (IACUC), as well as the following animal research statutes: Reporting of In Vivo Experiments (ARRIVE), EU Directive 2010/63/EU for animal experiments, and National Research Council’s Guide for the Care and Use of Laboratory Animals guidelines, and the procedures described in the Mexican Official Norm (NOM-062-ZOO-1999, modified in 2001) [60], revised in 2023 and entitled “Technical specifications for the production, care and use of laboratory animals”. The project experimental protocol was approved by the Research Ethics Committee of the National School of Biological Sciences (ENCB), with registration number CEI-ENCB-ZOO-02-2022.

#### 4.5.2. Experimental Diabetes Induced with STZ

Five days after acclimatization, initial blood glucose was measured with puncture of the caudal vein after a 6 h prior fasting period using Roche^®^ Accu-Chek^®^ test strips for glucose analysis (2019-11, lot 24686432; and 2020-04, lot 24692931, Hoffmann-La Roche, Paris, France) and Roche^®^ active glucometer (Item 42030003, UPC 4015630082988, Hoffmann-La Roche, Paris, France). Experimental diabetes-induced rats that were used as control groups (1 and 2, n = 6 each one) only received a single dose of citrate buffer (vehicle, 10 mM, pH 4.5, No. cat C2488, Sigma–Aldrich Chem. Co., St. Louis, MO, USA) at 2 mL/kg BW via intraperitoneal route (i.p.) [(1) Vehicles for healthy animals, (2) Control E200, healthy animals administered with MECP 200 mg/kg BW)]; remaining groups (3–6) were injected with single doses of STZ (No. cat S0130, Sigma–Aldrich Chem. Co., St. Louis, MO, USA) at 65 mg/kg BW, dissolved in citrate buffer (10 mM, pH 4.5) via same administration route [6,33,61]. After 72 h of single STZ injection, fasting blood glucose (FBG) level was quantified in all groups, and rats with a blood glucose concentration of ≥300 mg/dL were considered diabetic and granted entry to this study, after which they were randomly divided into the following diabetes-untreated and diabetic-treated groups (n = 6): (3) diabetic group (DG), diabetic-untreated animals, (4) DGVE, diabetic animals + vitamin E 250 mg/kg BW, (5) DGE100, diabetic animals + MECP 100 mg/kg BW, and (6) DGE200, diabetic animals + MECP 200 mg/kg BW. Treatments were immediately administered via intragastric (i.g.) route daily for six weeks every 24 h using a rigid oral cannula. FBG levels were measured every three weeks and total BW was registered every two weeks.

Two days prior to the end of the experiment, all experimental animals were placed in metabolic cages for 24 h to measure water and food consumption, as well as to collect and register urinary volume. Urine samples were reserved in freezing −4 °C for latter biochemical evaluations.

Rats were anesthetized with pentobarbital (45 mg/kg) via i.p. route, and a laparotomy process was performed to obtain blood samples from right renal artery without anticoagulant to obtain serum and latter kidneys tissues. Right kidneys were decapsulated, weighed, and separated into cortex and marrow to be stored in an ultra-freezing fridge at –80 °C.

##### Determination of Renal Relative Weight and Protein/DNA Ratios

Right kidney was weighted to calculate organ weight/BW ratio in mg both measures; after this, a sample of 500 mg was homogenized as described in the technique to measure protein/DNA ratio with TRIzol reagent method (Invitrogen, Grand Island, New York, NY, USA, TRIzoq, No. cat 15596-018, lot 50563207) according to Segura-Cobos et al. [33] and Amato et al. [62].

##### Kidney Function Evaluation

Proteinuria was quantified with modified Bradford method [63] using bovine serum albumin (BSA) (No. cat A2153, Sigma–Aldrich Chem. Co., St. Louis, MO, USA). A calibration curve was performed using BSA as a standard in a range of concentrations from 1 to 10 µg/mL (R^2^ = 0.98, y = 0.1296x + 0.0355). Results were showed as urinary total protein loss/24 h (mg/24 h).

Rats’ serum creatinine was measured according to kit instructions with fixed time colorimetric kinetic method. A calibration curve was performed using creatinine as a standard in a range of concentrations from 0.5 to 5 µg/mL (R^2^ = 0.97, y = 0.0296x − 0.0019) (Cayman Chemical, Ann Arbor, MI, USA). All absorbance was measured in a Shimadzu Double-Beam Scanning UV–Vis Spectrophotometer (Model UV-1700, SHIMADZU Corporation, Nishinokyo Kuwabara-cho, Nakagyo-ku, Kyoto 604-8511, Japan).

##### Enzymatic Antioxidant Activity and OS Biomarkers

- Tissue preparation

Renal cortex (100 mg) was homogenized in Eppendorf tubes with 1 mL of cold phosphate buffer (50 mM, pH 7) and a protease inhibitor mixture (Complete Mini Tablets, Roche). One tablet per each 10 mL of buffer solution [reference number SKU 11697498001, Sigma–Aldrich Chem. Co., St. Louis, MO, USA] was added at 4 °C and 10,000 r.p.m., in three cycles. Afterwards, 1 mL of each homogenate were centrifuged at 10,500 r.p.m. and 4 °C/15 min, and supernatant´s aliquots for each evaluation were subsequently taken. The rest of volume of complete uncentrifuged homogenates were kept at −80 °C until further use.

- OS evaluation

For antioxidant enzymes activity determination, all of absorbance was measured in a Shimadzu Double-Beam Scanning UV–Vis Spectrophotometer (Model UV-1700, SHIMADZU Corporation, Nishinokyo Kuwabara-cho, Nakagyo-ku, Kyoto 604-8511, Japan). All chemical reagents used were obtained from Sigma–Aldrich Company (St. Louis, MO, USA), unless otherwise indicated. Previous techniques described for catalase (CAT) [64], superoxide dismutase (SOD) [65] and glutathione peroxidase (GSH-Px) [66] were followed. Finally, Malonaldehyde (MDA), one of the end products of lipid peroxidation (LPO) reaction, was quantified in renal tissue also using Thiobarbituric acid reactive substances (TBARS) technique described by Ohkawa et al. [67].

#### 4.5.3. Statistical Analysis

GraphPad Prism ver. 8 software was utilized for analysis of results and graphic elaboration. Data were presented as standard error of the mean (SEM, ±) for quantitative results. Multiple comparisons between groups were performed using one-way analysis of variance (ANOVA) followed by Tukey’s post hoc test; values of *p* < 0.05 were considered statistically significant.

## 5. Conclusions

Results of this project indicated that the *Chiranthodendron pentadactylon* flower extract and its constituents, as promising supplements or nephroprotective agents, respectively, restored the renal function in rats with experimental diabetes in the treatment on complications derived from diabetes such as nephropathies, mainly through their antioxidant mechanism stimulating endogenous enzymatic defense and preventing lipid peroxidation in kidney tissues.

## Figures and Tables

**Table 1:** Effect of MECP on glycemia, body weight, food and water consumption and urinary volume after 6 weeks of treatment in diabetic rats.

Variable	Vehicle	DG	E200	DGVE	DGE100	DGE200
**Plasma glucose (mg/dL)**	79 ± 4.63	481.67 ± 11.61 *	103.5 ± 3.03	460.33 ± 17.37 *	497 ± 25.01 *	537 ± 19.73 *
**Body weight (g)**	369.5 ± 10.99	254.83 ± 11.12 *	355.17 ± 10.56	296.5 ± 18.29 *	277.33 ± 15.58 *	260.33 ± 7.84 *
**Food intake (g/24 h)**	10.54 ± 2.65	40.01 ± 1.87 *	5.64 ± 1.75	37.28 ± 1.36 *	35.59 ± 1.50 *	36.24 ± 2.29 *
**Water intake (mL/24 h)**	24 ± 3.03	146.67 ± 8.41 *	31.5 ± 5.49	152.83 ± 5.32 *	166.63 ± 7.88 *	149.5 ± 6.29 *
**Urinary volume (mL/24 h)**	8.08 ± 1.1	89 ± 5.54 *	14.17 ± 4.37	91.33 ± 7.64 *	111.17 ± 5.18 *	92.67 ± 5.72 *

Data are shown as mean ± SEM (n = 6). Analyzed with ANOVA followed by a Tukey post hoc test with * statistical significance (*p* < 0.05) vs. vehicle group; MECP, methanolic extract of *C. pentadactylon*; E200, healthy animals + MECP 200 mg/kg BW; DG, diabetic-untreated animals; DGVE, diabetic animals + vitamin E 250 mg/kg BW; DGE100, diabetic animals + MECP 100 mg/kg BW; DGE200, diabetic animals + MECP 200 mg/kg BW.

## Data Availability

The data presented in this study are available upon request from the corresponding author.
